# Diseases of the Kidney

**Published:** 1905-03-11

**Authors:** 


### Progress in Medicine and Surgery.

### DISEASES OF THE KIDNEY.

(*Continued from page* 409.)

Hydatid Disease—W. D. Wright[6] describes an instance of this rare condition in a man who had never been out of England. A swelling, gradually increasing in size, had been noticed below the ribs on the right side for seven years. Frequent attacks of jaundice had occurred for two or three years. The tumour, which was movable, and moved with respiration, reached to within an inch of the umbilicus and $1\frac{1}{2}$ inch of the intertubercular plane. No thrill could be made out, but fluctuation was noticed in the centre of the tumour. The abdomen was opened and an enlarged kidney was found. After an attempt at aspiration hydatid cysts appeared. The wound was then carefully packed to shield the general peritoneum and the mother cyst was washed out. The anterior and posterior layers of the peritoneum were held together and the kidney and hydatid were dissected out. The two layers of the peritoneum were sewn together, and the kidney cavity was plugged with gauze and drained. The patient, however, sank two days later. In Allbutt's "System of Medicine" it is noted that these tumours very often burst and discharge into the ureter, causing renal colic as the small cysts and products of suppuration pass away. The prognosis in such cases is good, though the trouble may last for years. There is frequently no thrill to be felt, and diagnosis is only possible when cysts are voided in the urine. Sir W. Roberts collected 63 instances of hydatids of the kidney, but in this country they are very seldom found.

Bilharziosis.—Sandwith[7] gives a full account of this plague which has appeared in many soldiers who have returned from South Africa. It is found in Lower Egypt in one-third of the population, and is widely spread elsewhere in Africa and Western Asia, and has recently been found twice in the United States[8] and in the West Indies. The worms are of two sexes and the male carries the female in a groove on the ventral side of the body. The eggs are found in thousands in the tissues of the human body and are voided in the urine and fæces. Those in the last situation have sometimes a lateral spine instead of a terminal one. The eggs do not hatch in any fluid of the body, but in fresh water this takes place in five minutes if the temperature is 86°. The life history of the embryo is unknown, and it has yet to be determined whether it usually enters the body through the mouth or the skin. There is a native belief that it enters the human urethra, which is also doubtful, but gets some apparent support from the enormous preponderance of the disease in males, especially in boys who bathe in the rivers. When it has effected an entrance it develops into a cyst, from which male and female worms arise after some months. The first symptom is a little blood passed at the end of micturition, this is increased after any excess of eating, drinking or exertion. There is no pain except a little backache or pricking in the penis, but cystitis, with papillomata, ulceration of the bladder, and tenesmus may follow, or similar troubles in the rectum. Very slight anæmia usually occurs, with generally an increase of eosinophile cells. Calculi and perineal fistulæ are among the complications, and recently a form of hepatic cirrhosis has been found to be due to the infection. The prognosis varies with the amount of infection ; the disease from a single infection seems to die out in three years and some patients may have little trouble from it, but in others death may take place from accumulated bladder and rectal troubles. The only drug which has any effect is 15 drops of the extract of male fern three times a day which should not be continued for more than a fortnight at a time. Hæmostatic injections for the hæmaturia are harmful, but early cystitis may be relieved by washing out the bladder with boroglyceride, or by salol and urotropin.

[6] Lancet, Jan. 21. [7] Pract., Oct. [8] Med. Rec., June.

*(To be continued.)*

